# A Case Report on Iris Disk Positioning on a Custom-Made Ocular Prosthesis Using an Adjustable Trial Frame

**DOI:** 10.7759/cureus.56382

**Published:** 2024-03-18

**Authors:** Sadhvi G Naik, Muness Akhtarkhavari, Aradhana Nagarsekar, Meena A Aras, Vidya Chitre

**Affiliations:** 1 Prosthodontics and Crown and Bridge, Goa Dental College and Hospital, Bambolim, IND

**Keywords:** iris positioning, adjustable trial frame, customised impression tray, custom ocular prosthesis, ocular defect

## Abstract

Trauma, cancer, and congenital defects may all result in the loss of an eye. This leads to significant emotional and physical challenges in an individual’s life. In such cases, cautious preoperative, surgical, and prosthetic planning employing a multidisciplinary approach is essential for effective rehabilitation. Iris positioning is one of the crucial steps in the fabrication of a customized ocular prosthesis. Iris positioning is a technique-sensitive process, hence visual evaluation by itself could not provide reliable results. This case report illustrates a method of iris disk positioning on a custom-made ocular prosthesis using an adjustable trial frame. The advantage of the adjustable mechanism of the adjustable trial frame was utilized here to position the iris on the scleral blank. Since the iris disk on the ocular prosthesis was positioned in symmetry with that of the natural eye, the patient's aesthetics were restored.

## Introduction

Partial or complete loss of an eye affects the patient's ability to see and results in an obvious facial deformity [1]. The main objective of treating an acquired or congenital eye defect with an aesthetically acceptable prosthesis is to let the patient confront the outside world and resume their regular activities [2]. The precise positioning of the iris on the custom-made ocular prosthesis is one of the most difficult aspects of the fabrication of the prosthesis [3]. Iris positioning is important for obtaining the accurate interpupillary distance and its orientation in relation to the natural eye. Numerous techniques for accurately placing the iris disk on the custom-made ocular prosthesis have been developed. This article reports a case in which an iris disk was positioned on a custom-made ocular prosthesis using an adjustable trial frame.

This article was presented as a poster at the 50th Conference of the Indian Prosthodontic Society on November 11, 2022, in New Delhi.

## Case presentation

**Figure 1:**
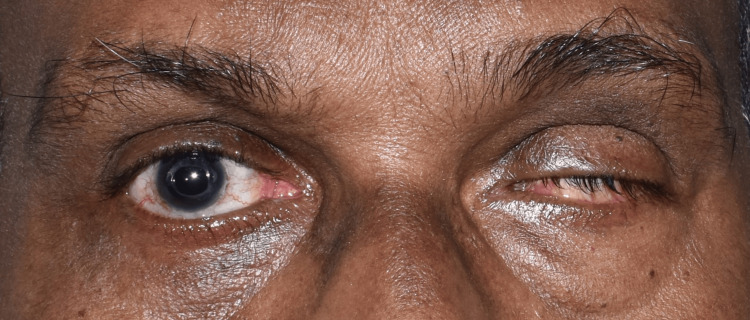
Pretreatment photograph

A 65-year-old man came to the prosthodontics crown and bridge department in order to get his missing left eye prosthetically restored. On enquiring about the patient's past, it was discovered that a year prior, he had suffered a catastrophic injury to his left eye that required surgical evisceration of the eye (Figure 1).

**Figure 2:**
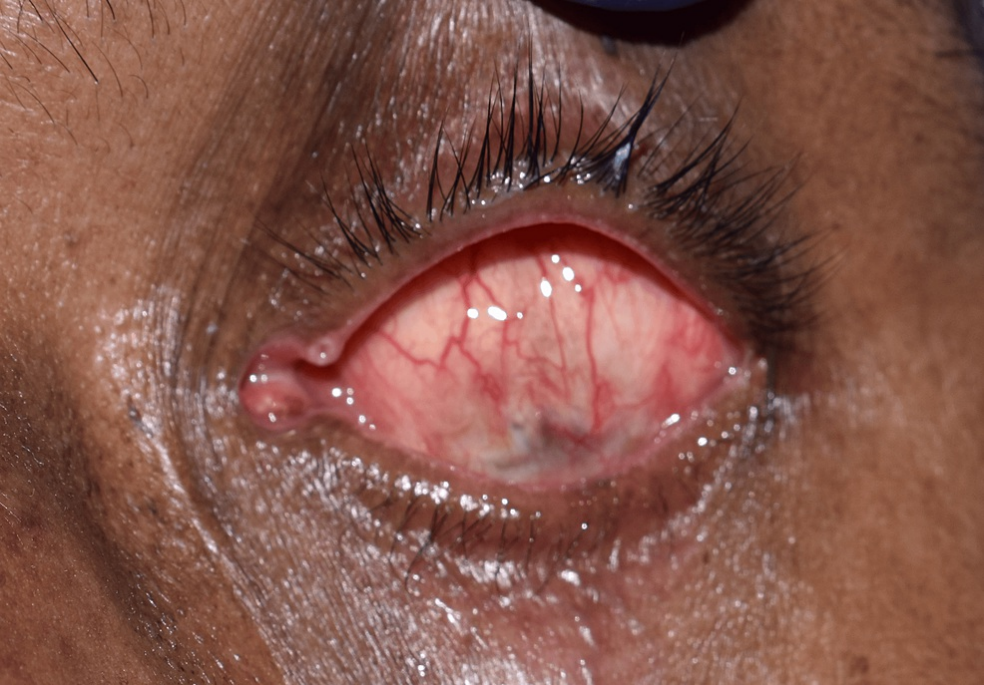
Eviscerated left eye socket

**Figure 3:**
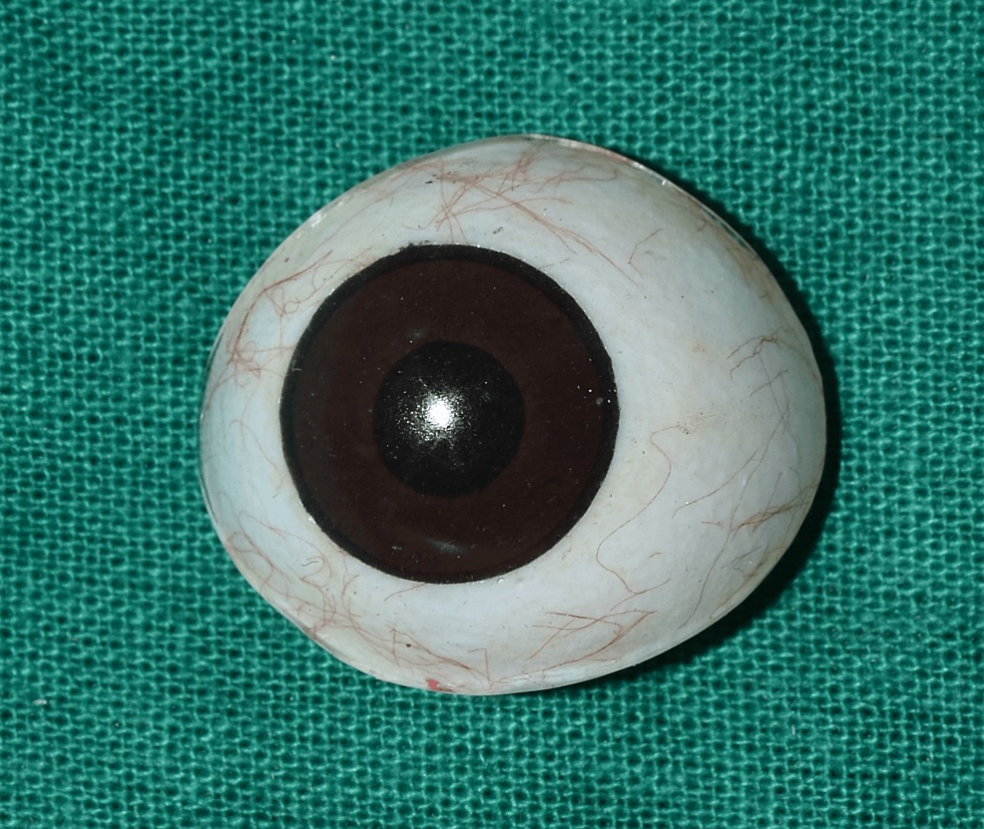
Stock eye with matching iris

**Figure 4:**
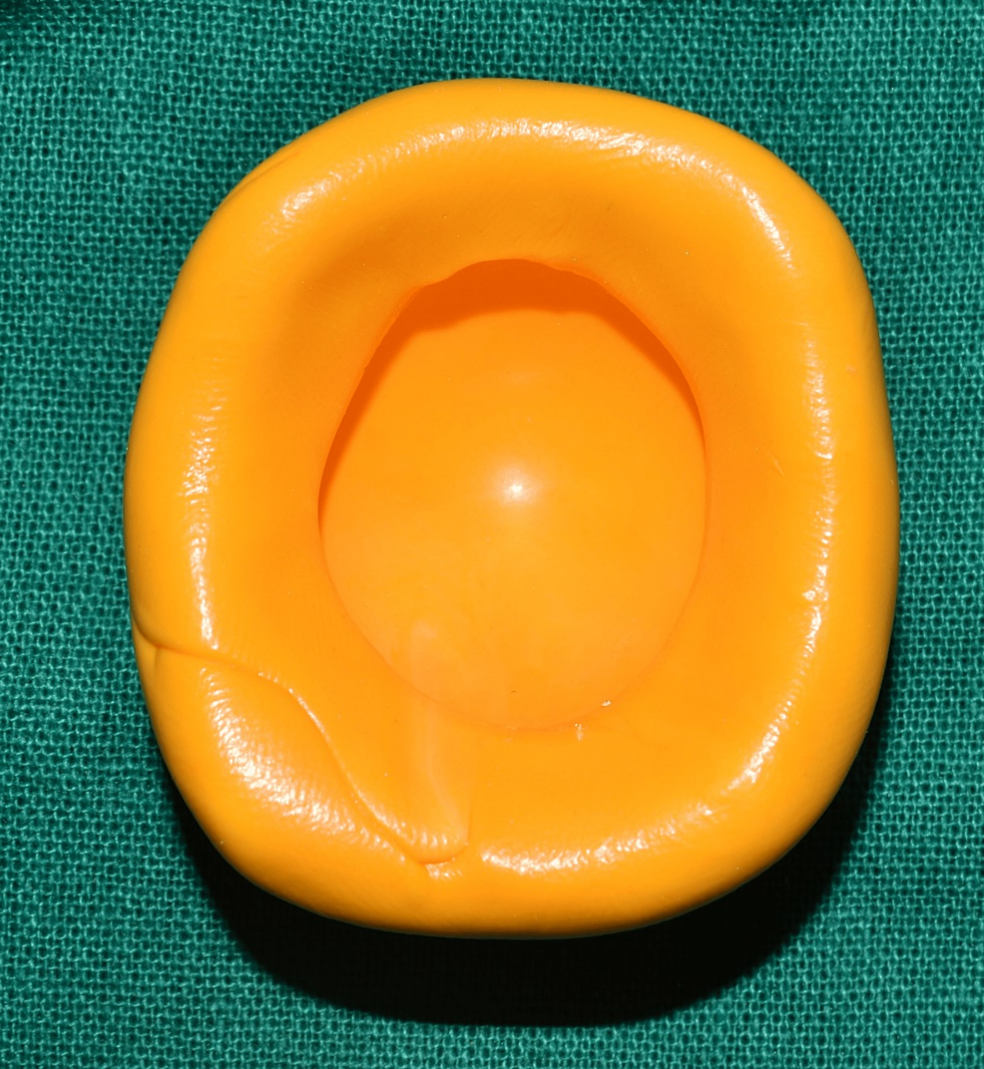
Putty impression made using the stock eye

**Figure 5:**
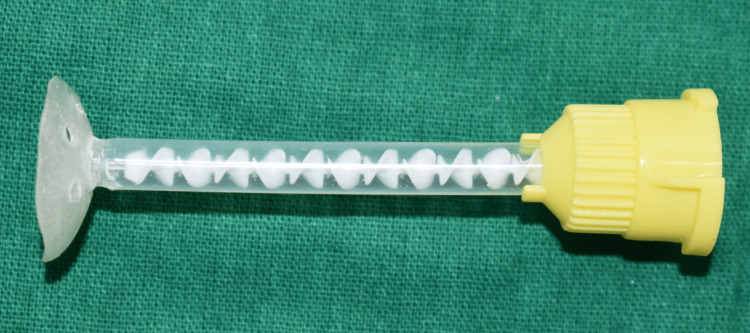
Custom tray with the auto-mixing tip

**Figure 6:**
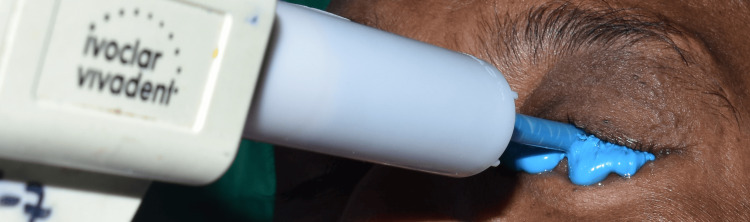
Impression making

**Figure 7:**
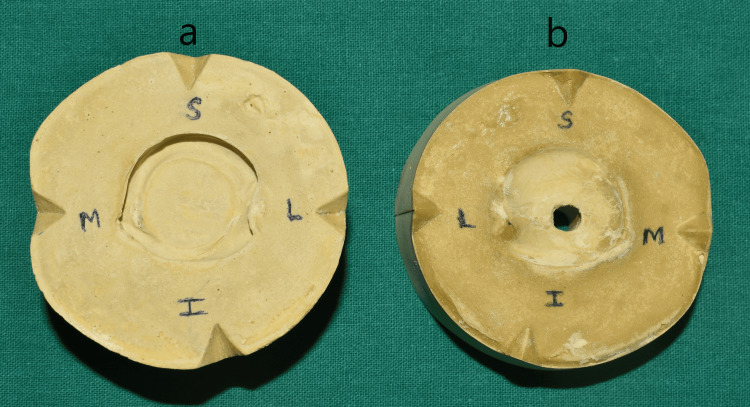
Mold obtained using the two-pour split cast technique a: cavity of the mold, b: core of the mold

On evaluation, the tissue bed was in good shape, the eyelids were unharmed, and the space between the upper and lower eyelids was deep enough to accommodate an ocular prosthesis (Figure 2). No gross facial asymmetry was noted. The Department of Ophthalmology had referred the patient with a stock eye with matching iris (Figure 3). Informed consent was obtained from the patient, and it was decided to make a customized ocular prosthesis utilizing the stock eye's iris. To make a mold, a putty impression (Zhermack Elite HD+; Zhermack GmbH, Badia Polesine (Rovigo), Italy) was made using the stock eye (Figure 4). A clear auto-polymerizing acrylic resin was used for making a customized tray in the putty mold (DPI, India). In the socket, the tray's margins were modified, and vent holes were created. This tray had an auto-mixing tip attached to it (Figure 5) that facilitated the flow of light body addition silicone (Aquasil Dentsply) to record the tissue bed (Figure 6). The impression was poured using the two-pour split cast technique (type III dental stone) to obtain a mold (Figure 7).

**Figure 8:**
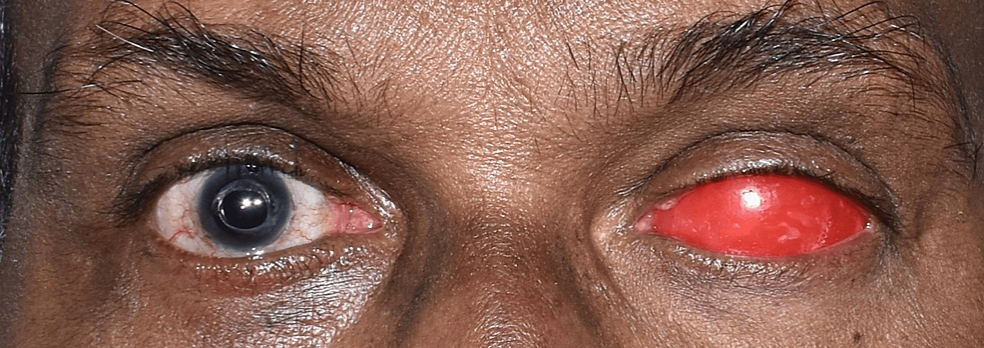
Wax pattern trial

This mold was filled with modeling wax (Deepti Dental Products of India Pvt. Ltd., Delhi, India) to create a wax pattern, which was then adjusted on the patient to achieve an appropriate shape (Figure 8). The investing of the wax pattern was followed by dewaxing. A clear heat-polymerizing acrylic resin and teeth molding powder were used to fabricate a scleral blank (DPI, India). The patient's glabella, tip of the nose, chin, and forehead crease were used to mark the midline for iris positioning. The patient was asked to align the opposing eye by looking off at the distant point after the scleral blank had been inserted into the socket.

**Figure 9:**
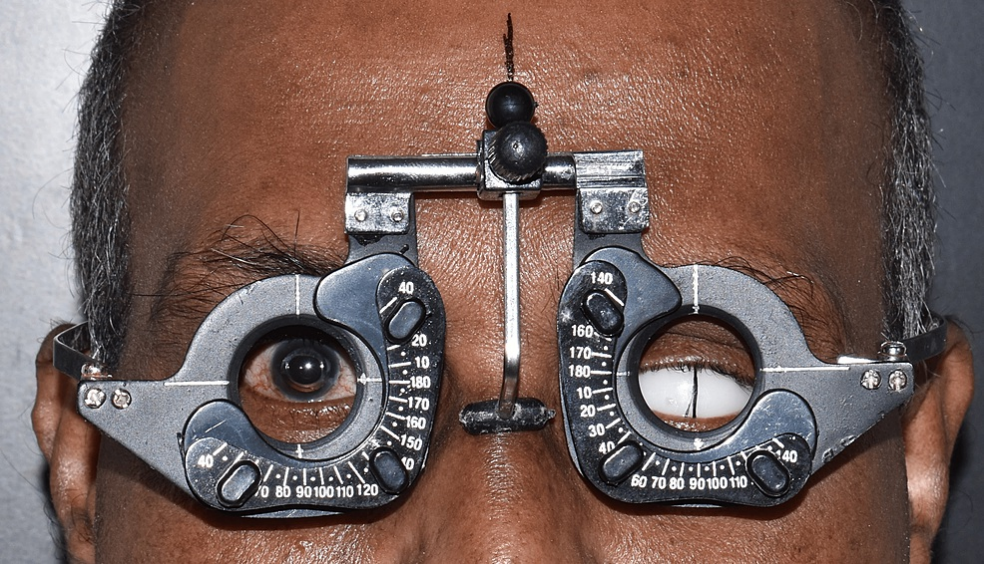
Iris positioning on the scleral blank using the adjustable trial frame

**Figure 10:**
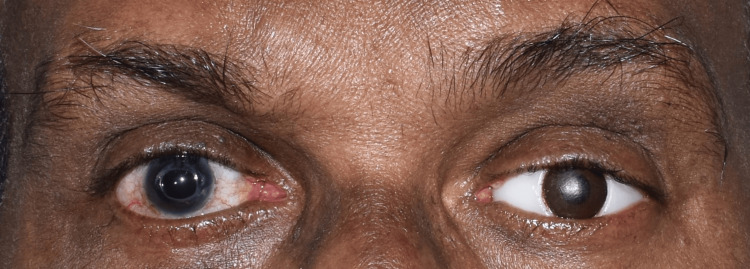
Iris position verified

The location of the iris was then marked on the scleral blank using an adjustable trial frame (Medik Trial Frame, Regular, Eye Care Products, Delhi, India) with respect to the inner and outer canthus and the upper and lower eyelids of the normal contralateral eye (Figure 9). Following this, the iris from the stock eye was separated and placed on the scleral blank using cyanoacrylate glue, and its placement was verified (Figure 10).

**Figure 11:**
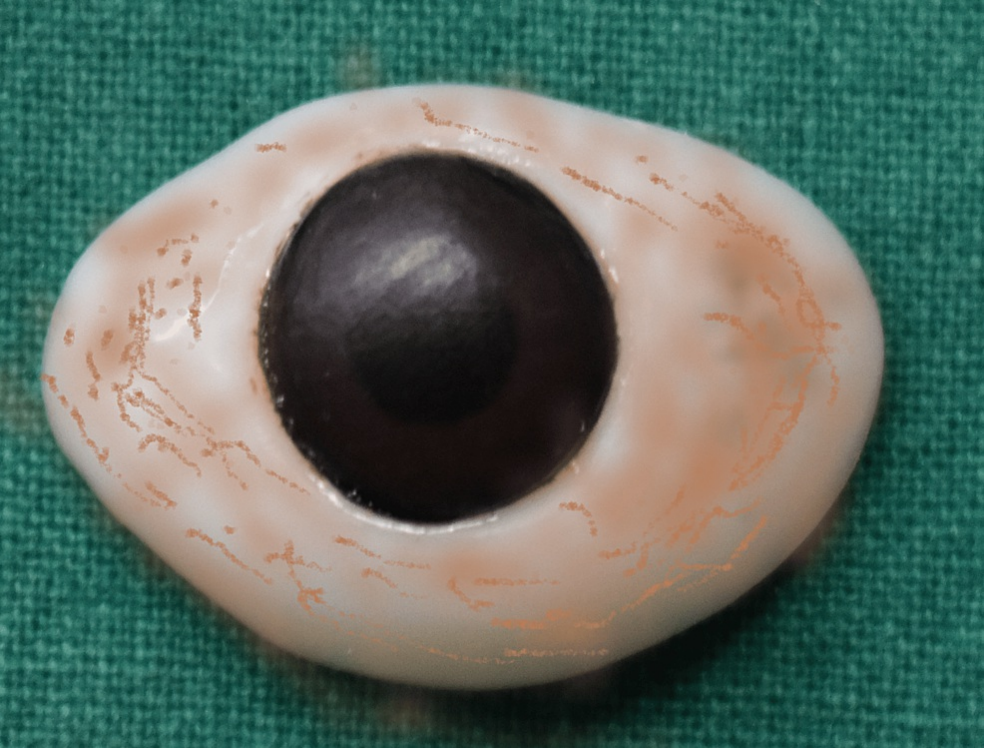
Final prosthesis

**Figure 12:**
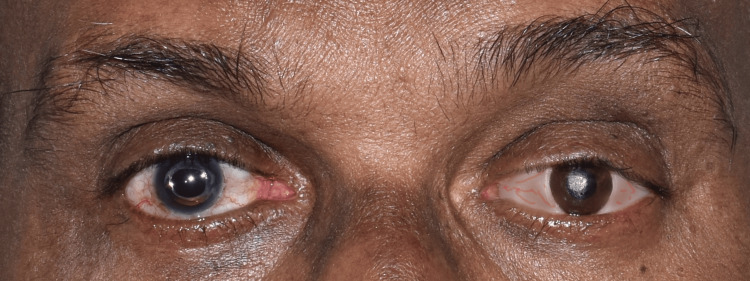
Prosthesis in situ

Composite paints (SR Adoro Stains, Ivoclar Vivadent, Germany) were used to tint the sclera, and it was cured in the polymerization unit. The stains on the prosthesis were first photoactivated for 40 seconds in accordance with manufacturer instructions (Quick Curing Unit, Ivoclar Vivadent, Liechtenstein). The polymerization of the prosthesis was then finished by placing it in the Targis Power Upgrade unit under light and vacuum for 16 minutes. To preserve the characterization, a layer of protection (G-Coat Plus, GC America Inc., Alsip, IL, USA) was put on. The final prosthesis with post-delivery instructions was delivered to the patient (Figures 11, 12). The patient was evaluated after a day, week, and month. The patient was content with the ocular prosthesis.

## Discussion

Surgical intervention may be needed for congenital defects, accidental trauma, or pathologies that may result in the removal of the eyeball [4,5]. Peyman GA et al. [6] divided eye removal surgical techniques into three groups: the removal of the globe's contents while keeping the sclera, extraocular muscles, and optic nerve intact is known as Evisceration. When the muscles and optic nerve are severed, the globe is removed completely, a process known as Enucleation and Exenteration that involves removing all tissues from the orbit and the socket, including the globe, conjunctiva, orbital fat, and all or part of the eyelids [7,8]. An ocular prosthesis is fabricated for the first two surgeries, and an orbital prosthesis is given for the third [7]. A customized ocular prosthesis requires an impression of the socket, a wax pattern trial, iris positioning, and acrylization [9]. The most crucial stage in the fabrication of a customized ocular prosthesis is iris positioning [3]. Different techniques and customized instruments have been reported in the literature for positioning the iris accurately in a prosthetic eye from 1969 until 2019 (Table 1) [10].

**Table 1:** Studies regarding iris positioning in the ocular prosthesis *Credit: Positioning of iris in an ocular prosthesis: A systematic review; Sathe et al. [10] CAD: computer-aided design, CAM: computer-aided manufacturing, IPD: interpupillary distance

Study	Year	Technique/Instrument
Roberts et al [11]	1969	Pupillometer
Brown et al [12]	1970	Facial measurements using anatomic landmarks
Joneja OP et al [13]	1976	Window light
Helene James et al [14]	1976	Visual assessment
McArthur et al [15]	1977	Ocular locator
Nusinov et al [16]	1988	Inverted anatomic tracings
Guttal et al [8]	2007	Graph grid method
Manvi S et al [17]	2008	Boleys gauge
Pai et al [3]	2010	Grid cutouts placed on the spectacle frame
Gupta et al [18]	2013	Customized scale
Yunpen Bi et al [19]	2013	CAD/CAM
Shetty PP et al [20]	2017	Modified Hanau wide‑view spring bow
Chamaria et al [9]	2017	Customized frame spring bow assembly
Bhochhibhoya et al [21]	2019	Pupillary distance ruler
Dasgupta et al [22]	2019	Digital photograph
Chihargo et al [23]	2019	Optical vernier IPD ruler
Lanzara et al [24]	2019	Electronic vernier caliper

In this case report, iris positioning on a custom-made ocular prosthesis was done using an adjustable trial frame. This frame is used by ophthalmologists to hold the lenses and other accessories for assessing refractive errors [25]. There are knobs to adjust the interpupillary distance, side angle, and height. The positions of the two side assemblies give an exact measurement of the interpupillary distance [26]. Iris positioning was carried out on the scleral blank using this adjustable mechanism in relation to the upper and lower eyelids, as well as the inner and outer canthus, of the normal contralateral eye. The technique presented here has shown positive outcomes in terms of patient aesthetics, acceptability, and satisfaction.

Other advantages of this technique include the fact that it requires only one armamentarium, which is readily available, requires less expertise due to its simplicity, allows for repeated verification of iris position, and can be used on multiple patients.

However, accurate identification of the midline and margins of the intact iris may lead to subjective errors in cases of face asymmetry. Patients without ears cannot utilize this technique, as stabilization of the adjustable trial frame is necessary to attain accuracy in iris disk positioning.

## Conclusions

The ocular and orbital prostheses are no exception to the general principle that symmetry is the key to the pleasing esthetics of maxillofacial prostheses. Several methods have been evolved to make ocular and orbital prostheses that restore aesthetic function and boost patient confidence. The accurate positioning of the iris disc assembly results in a successful ocular and orbital prosthesis. The method outlined in this article utilizes the benefits of adjustability of the trial frame that helps us achieve an accurate registration of the iris disk position, mirroring a natural look.
